# Empowering PET imaging reporting with retrieval-augmented large language models and reading reports database: a pilot single center study

**DOI:** 10.1007/s00259-025-07101-9

**Published:** 2025-01-23

**Authors:** Hongyoon Choi, Dongjoo Lee, Yeon-koo Kang, Minseok Suh

**Affiliations:** 1 Department of Nuclear Medicine, Seoul National University Hospital, 101 Daehak-ro, Jongno-gu, Seoul, 03080 Republic of Korea; 2 Department of Nuclear Medicine, Seoul National University College of Medicine, Seoul, Republic of Korea; 3 Portrai, Inc., Seoul, Republic of Korea

**Keywords:** PET reports, Large language model, Retrieval-augmented generation, Artificial intelligence

## Abstract

**Purpose:**

The potential of Large Language Models (LLMs) in enhancing a variety of natural language tasks in clinical fields includes medical imaging reporting. This pilot study examines the efficacy of a retrieval-augmented generation (RAG) LLM system considering zero-shot learning capability of LLMs, integrated with a comprehensive database of PET reading reports, in improving reference to prior reports and decision making.

**Methods:**

We developed a custom LLM framework with retrieval capabilities, leveraging a database of over 10 years of PET imaging reports from a single center. The system uses vector space embedding to facilitate similarity-based retrieval. Queries prompt the system to generate context-based answers and identify similar cases or differential diagnoses. From routine clinical PET readings, experienced nuclear medicine physicians evaluated the performance of system in terms of the relevance of queried similar cases and the appropriateness score of suggested potential diagnoses.

**Results:**

The system efficiently organized embedded vectors from PET reports, showing that imaging reports were accurately clustered within the embedded vector space according to the diagnosis or PET study type. Based on this system, a proof-of-concept chatbot was developed and showed the framework’s potential in referencing reports of previous similar cases and identifying exemplary cases for various purposes. From routine clinical PET readings, 84.1% of the cases retrieved relevant similar cases, as agreed upon by all three readers. Using the RAG system, the appropriateness score of the suggested potential diagnoses was significantly better than that of the LLM without RAG. Additionally, it demonstrated the capability to offer differential diagnoses, leveraging the vast database to enhance the completeness and precision of generated reports.

**Conclusion:**

The integration of RAG LLM with a large database of PET imaging reports suggests the potential to support clinical practice of nuclear medicine imaging reading by various tasks of AI including finding similar cases and deriving potential diagnoses from them. This study underscores the potential of advanced AI tools in transforming medical imaging reporting practices.

**Supplementary Information:**

The online version contains supplementary material available at 10.1007/s00259-025-07101-9.

## Introduction

The integration of Large Language Models (LLMs) into the clinical domain has heralded a new era in healthcare innovation, particularly in the realm of medical imaging reports [1, 2]. LLMs, with their sophisticated zero-shot learning capabilities, have shown promise in parsing, summarizing, and generating complex medical texts, thereby enhancing the efficiency and accuracy of clinical documentation and decision-making processes [3]. Their application extends across various specialties, aiming to revolutionize how healthcare professionals interact with and leverage vast amounts of medical data for patient care.

Despite the growing interest and proven benefits of LLMs in many areas of medicine, their potential has not been fully explored in the realm of nuclear medicine imaging, particularly PET imaging reporting. Despite the potential of ChatGPT to revolutionize content creation by generating human-like text [4], specific applications leveraging LLMs in the nuclear medicine field, particularly for imaging reports, has not been explored. PET imaging, which is performed for a variety of purposes and conditions, produces complex data requiring thorough analysis and interpretation, playing a critical role in clinical decision-making [5, 6]. There is a need for advanced tools to aid in referencing past reports, sourcing cases for educational purposes, and conducting differential diagnoses, especially as the use of PET, which encompasses various radiotracers and diseases, becomes more widespread. This unmet need presents a significant opportunity for LLMs to improve the specificity and relevance of PET report generation. By leveraging prior reports and analogous case studies, LLMs can provide clinicians with valuable insight, aiding them in making informed decisions.

In this study, we introduce a pioneering approach to PET imaging reporting by developing and accessing a custom-built, retrieval-augmented generation (RAG) LLM framework [7]. This system leverages a comprehensive large database of PET reading reports. By embedding these reports into a vector space for efficient retrieval based on similarity metrics, our framework aims to enhance PET imaging reporting in three key ways: (1) Assisting PET reading experts by referencing past reports, enabling them to review similar cases and outcomes during the diagnostic process. (2) Supporting educational purposes by identifying appropriate cases for case-centered study. (3) Facilitating interactive queries related to PET reading for clinicians, based on a database of past reports. This proof-of-concept study seeks to demonstrate the feasibility and benefits of integrating advanced LLM capabilities with a vast repository of PET imaging data, aiming to set enhanced medical imaging reporting practices.

## Materials and methods

### Dataset

This study was conducted at a single center, utilizing reading reports of PET imaging data sourced from the clinical data warehouse (CDW) of the SUPREME Platform. We extracted data spanning from 2010 to 2023, comprising reports from 118,107 patients across 211,813 cases. Institutional Review Board (IRB) approval was secured from our hospital (IRB No. 2401-090-1501), with the requirement for written informed consent waived due to the retrospective nature of the study and the use of deidentified information. The dataset encompassed reading reports for all cases, along with the exam date, exam name, a deidentified research identifier (ID), sex, and date of birth (year-month format).

### Model architecture

**Fig. 1:**
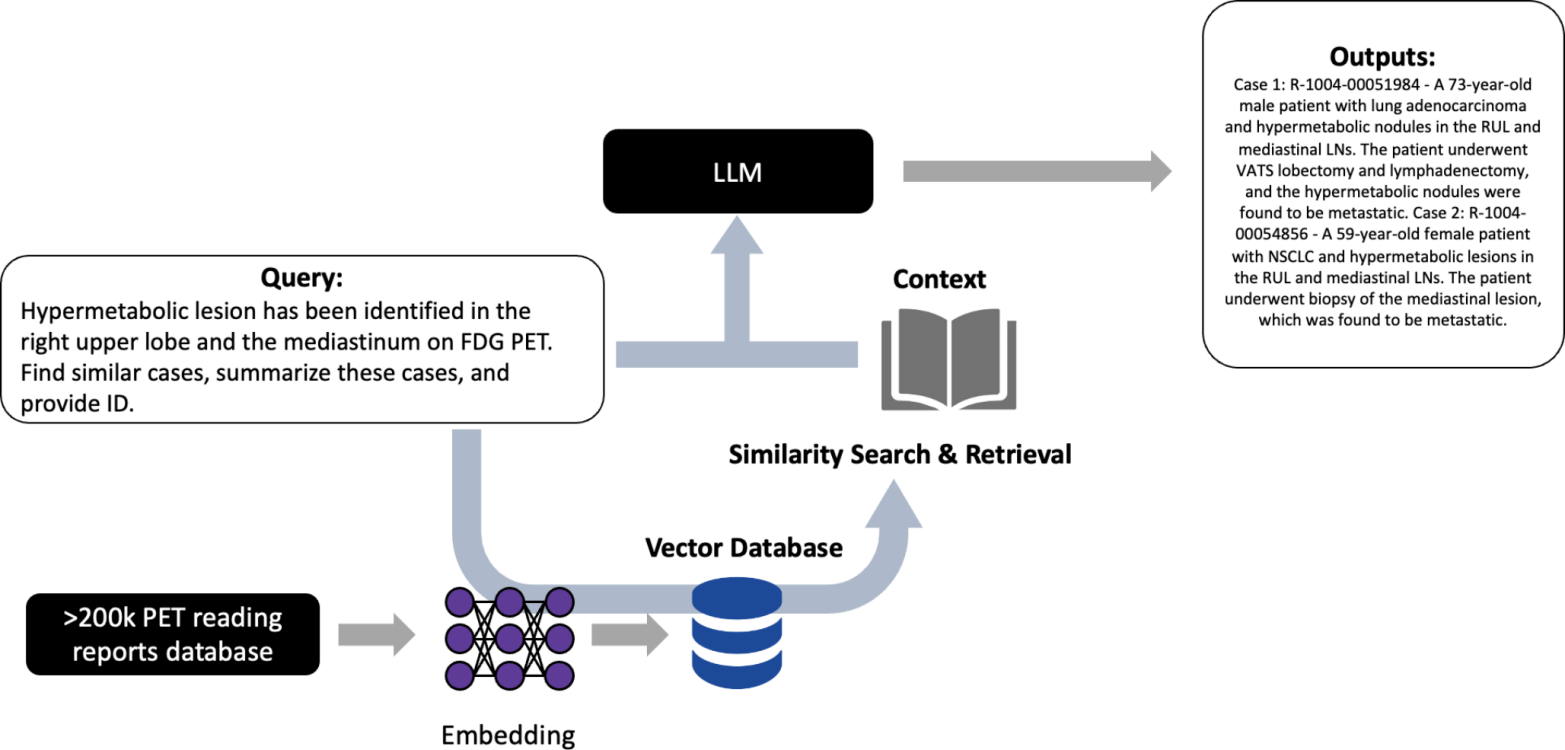
Workflow of the Chatbot System for Querying PET Imaging Reading Reports. The overall workflow of the proof-of-concept system designed for efficient querying of reading reports from a substantial dataset is illustrated. The system integrates the Retrieval-Augmented Generation (RAG) model with advanced language model technologies, natural language processing, and information retrieval techniques. The workflow demonstrates the process from user query input through to the delivery of the relevant reading report, showcasing the operational framework and interaction with different sources of reading reports

In this study, we designed a proof-of-concept chatbot system for efficiently querying reading reports from a substantial dataset. It was based on ‘RAG’ [7]. The adaptability of this system allows for the utilization of various database formats, including but not limited to ‘csv’ files, to accommodate different sources of reading reports. This system amalgamates state-of-the-art language model technologies with sophisticated natural language processing and information retrieval techniques, aiming to deliver precise, contextually relevant responses to inquiries concerning PET imaging reading reports. The overall workflow of this system is illustrated in Fig. 1.

The architecture of our system is underpinned by a series of modular components, each crucial for interpreting and responding to user queries. At the forefront is a sentence embedding layer, crafted to process intricate texts and queries by transforming sentences into vectors. This transformation facilitates subsequent processing by various mathematical models. We employed the Sentence Transformer model, specifically the “paraphrase-multilingual-MiniLM-L12-v2” (https://huggingface.co/sentence-transformers/paraphrase-multilingual-MiniLM-L12-v2), renowned for its ability to comprehend and paraphrase texts across multiple languages—a necessary feature considering the bilingual nature (English and Korean) of the reading reports in our dataset. To manage and retrieve PET reading reports effectively, our system incorporates a vector storage mechanism, Chroma (Chroma, https://www.trychroma.com/). Chroma organizes textual data into a searchable vector space by converting text into numerical vectors derived from the sentence embeddings. This conversion enables the system to execute advanced retrieval operations, identifying responses that are semantically relevant to the queries posed. The retrieval after embedding to Chroma was performed using the cosine similarity of the query text vectors, retrieving the top-k texts from the database as context for generating prompts for the LLM. We set this top-k value to k = 5.

After retrieving the related context, specifically previous PET reports, a question-answering (QA) component was integrated. This QA mechanism excels at comprehending user queries, sourcing the most pertinent documents from the dataset, and formulating informative responses that precisely address the queries. To generate prompts, the system integrates retrieved texts as contexts along with the reader’s question to create a full prompt. For example, the prompt includes the text: “*Give an answer by only referring to the context*,* include the address within the context in the answer*,* and clearly number the answer*,” along with (*context*), which contains the retrieved reports, and (*question*), representing the reader’s query. For the generation of these responses, we incorporated the Llama-3 (7-billion parameter model) language model [8] and the system architecture was based on Langchain [9].

### Visualization of vector embedding

Following the process of sentence embedding, the resulting vectors were stored in a vector database. These vectors played a crucial role in identifying similarities between various texts, including the queries submitted to the system. To facilitate a deeper understanding of how PET reading reports are represented within this vector space, we employed t-distributed Stochastic Neighbor Embedding (t-SNE) for visualization purposes [10]. Specific keywords associated with imaging reports, such as “lung cancer,” “breast cancer,” “lymphoma,” “methionine PET,” and “PSMA PET,” were chosen for this analysis. The objective was to ascertain whether reports containing these selected terms would naturally form distinct clusters within the vector space. This approach aimed to visually demonstrate the effectiveness of our vector embedding process in grouping similar reports, thereby providing insights into the semantic relationships and similarities between different PET reports in the dataset.

### Test examples

In the evaluation of prototype chatbots designed for navigating an extensive database of PET reading reports, we focused on testing their ability to accurately retrieve reports similar to those specified in user queries and to assist in differential diagnosis by referencing previous reports. This involved assessing the proficiency in identifying cases with specific diagnoses or imaging findings and their capability to extract relevant information to support nuclear medicine experts in diagnosing complex cases. The testing protocol simulated real-world scenarios, presenting the chatbots with diverse clinical questions to comprehensively evaluate their utility in clinical decision-making and their effectiveness in leveraging the vast database to enhance the accuracy and relevance of their responses.

### Evaluation of queried similar cases and potential diagnoses

From daily routine PET exams, we simulated prompts to evaluate relevance and appropriateness by three independent nuclear medicine physicians. We extracted 19 cases from routine PET exams and their reports to evaluate two tasks: query performance for similar cases and potential diagnoses from findings. To evaluate query performance for similar cases, we used the text from the conclusions of the PET reports to generate prompts such as “find similar cases and summarize the reports.” For evaluating potential diagnoses, specific texts from the findings sections of the reports were used to generate prompts to “suggest potential diagnoses for this finding.” Examples of conclusions and findings used for these prompts are summarized in Supplementary Table 1. Three nuclear medicine physicians independently scored the system’s answers for medical relevance on a scale of 1 (poor), 2 (fair), and 3 (good). The gold standard for these evaluations was the consensus judgment of these experienced physicians, who assessed the medical relevance and accuracy of the system’s responses based on their expert knowledge and clinical experience. To assess the effect of the RAG on the performance of the LLM, we compared the appropriateness scores of the LLM with and without RAG using the Wilcoxon rank-sum test. This comparative analysis helped determine the added value of the RAG framework in enhancing the relevance and accuracy of the generated responses.

In addition to performance evaluations based on physician scoring, a quantitative assessment was conducted to evaluate the accuracy of conclusions generated from findings. By inputting text from the findings section, the LLM with and without RAG was tested for its ability to generate conclusion texts for reading reports, simulating diagnostic reasoning. (prompt: “*Write a concise conclusion*,* including a potential diagnosis*,* in one or two sentences*”). These generated conclusions were compared to the actual conclusion reports described by nuclear medicine physicians. The comparisons were quantified using the ROUGE-L metric (Recall-Oriented Understudy for Gisting Evaluation), which measures the alignment between generated and reference texts by focusing on the longest common subsequences (LCS) while accounting for word order [11, 12]. To assess the overall quantitative performance, the ROUGE-L F-score—representing the harmonic mean of precision and recall—was calculated for both the LLM with RAG and without RAG. This evaluation highlights the impact of the RAG framework on improving the alignment and relevance of the generated conclusions.

## Results

### Clustered unstructured PET reports by sentence embedding

**Fig. 2:**
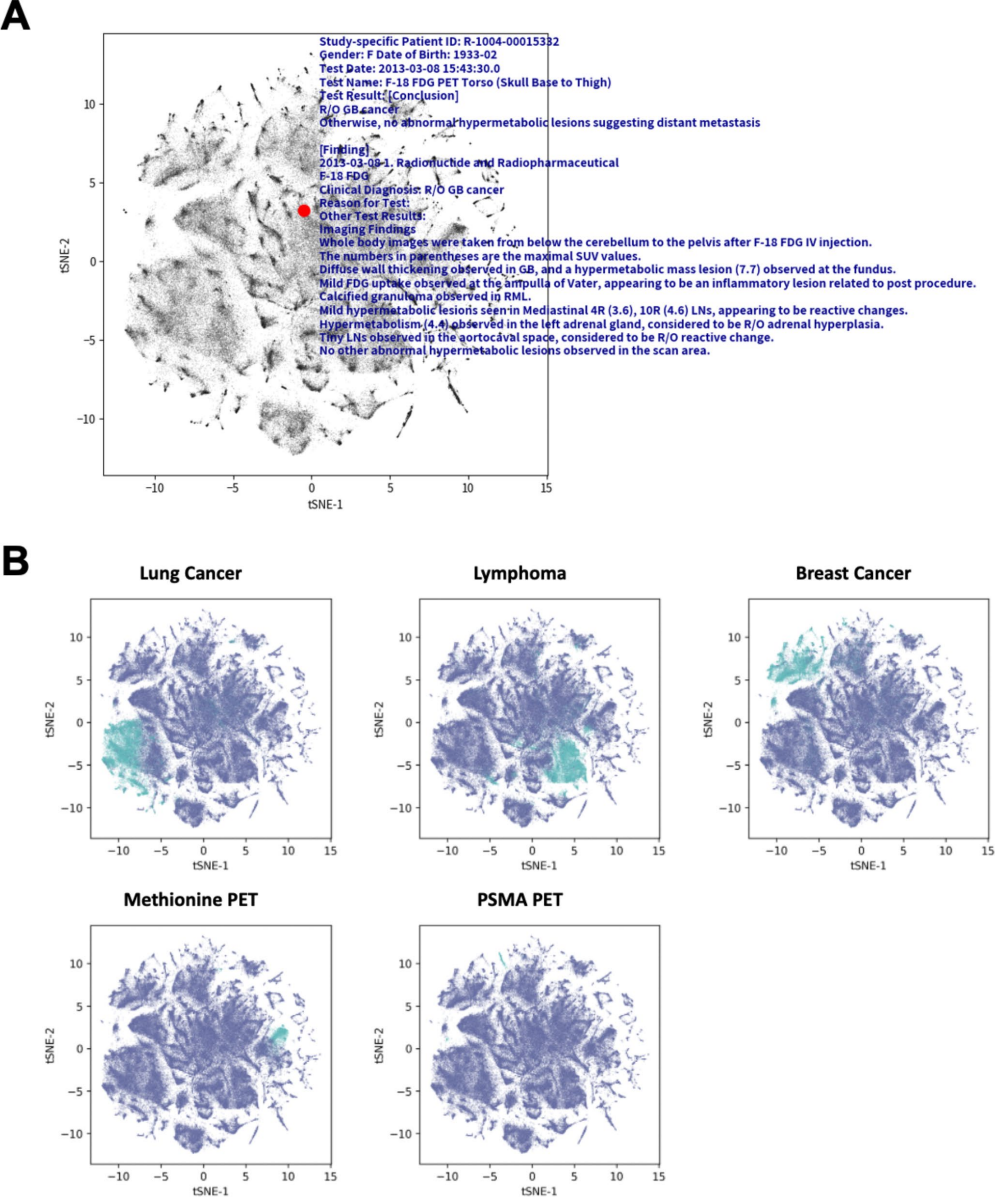
Visualization of PET Imaging Report Embeddings Using t-SNE. (**A**) t-SNE plot illustrates PET imaging report embeddings from 118,107 patients, totaling 211,813 cases. Each point on the plot represents a unique report, with a selected case highlighted in red to show an example of an original report. (**B**) t-SNE plots showcases the clustering efficacy of the embeddings, highlighting how reports containing key diagnostic terms like ‘lung cancer’, ‘breast cancer’, ‘lymphoma’, and specific types of exams such as ‘C-11 methionine PET’ and ‘Ga-68 PSMA-11 PET’ form distinct clusters. These clusters indicate the embeddings’ capability to reflect the similarity among cases, demonstrating the potential of this method in facilitating the identification and visualization of related PET imaging reports

We analyzed PET imaging reports from 118,107 patients, totaling 211,813 cases, by converting them into vector embeddings. These embeddings were then visualized on a t-SNE plot to demonstrate dimensionality reduction and the clustering of reports with similar characteristics (Fig. 2A). Each point on this plot represents a unique PET imaging report, with a specific case highlighted in red for illustrative purposes, including its original report. By examining the distribution of these clusters, we observed distinct groupings based on diagnostic terms and exam types, indicating that reports with similar clinical contexts naturally grouped together in the embedding space. For instance, to evaluate the representational efficacy of the embeddings, reports containing key diagnostic terms such as ‘lung cancer’, ‘breast cancer’, and ‘lymphoma’, as well as those pertaining to specific types of exams like ‘C-11 methionine PET’ and ‘Ga-68 PSMA-11 PET’, were marked on the plot. The clusters containing ‘lung cancer’ exhibited substantial cohesion, potentially reflecting the higher prevalence of lung cancer cases in our dataset, while distinct clusters also emerged for ‘breast cancer,’ ‘lymphoma,’ and specific PET modalities such as C-11 methionine PET and Ga-68 PSMA-11 PET (Fig. 2B). These cohesive clusters highlight clinically meaningful patterns, suggesting that sentence embeddings from unstructured reports could be leveraged to make a context for using LLM for question and answering. The formation of these distinct clusters underscores the text embedding ability in PET reports to reflect the semantic similarity among cases, offering potential clinical utility in identifying disease-specific patterns and retrieving relevant texts.

### LLM with RAG chatbot-assisted querying and suggested diagnosis

**Fig. 3:**
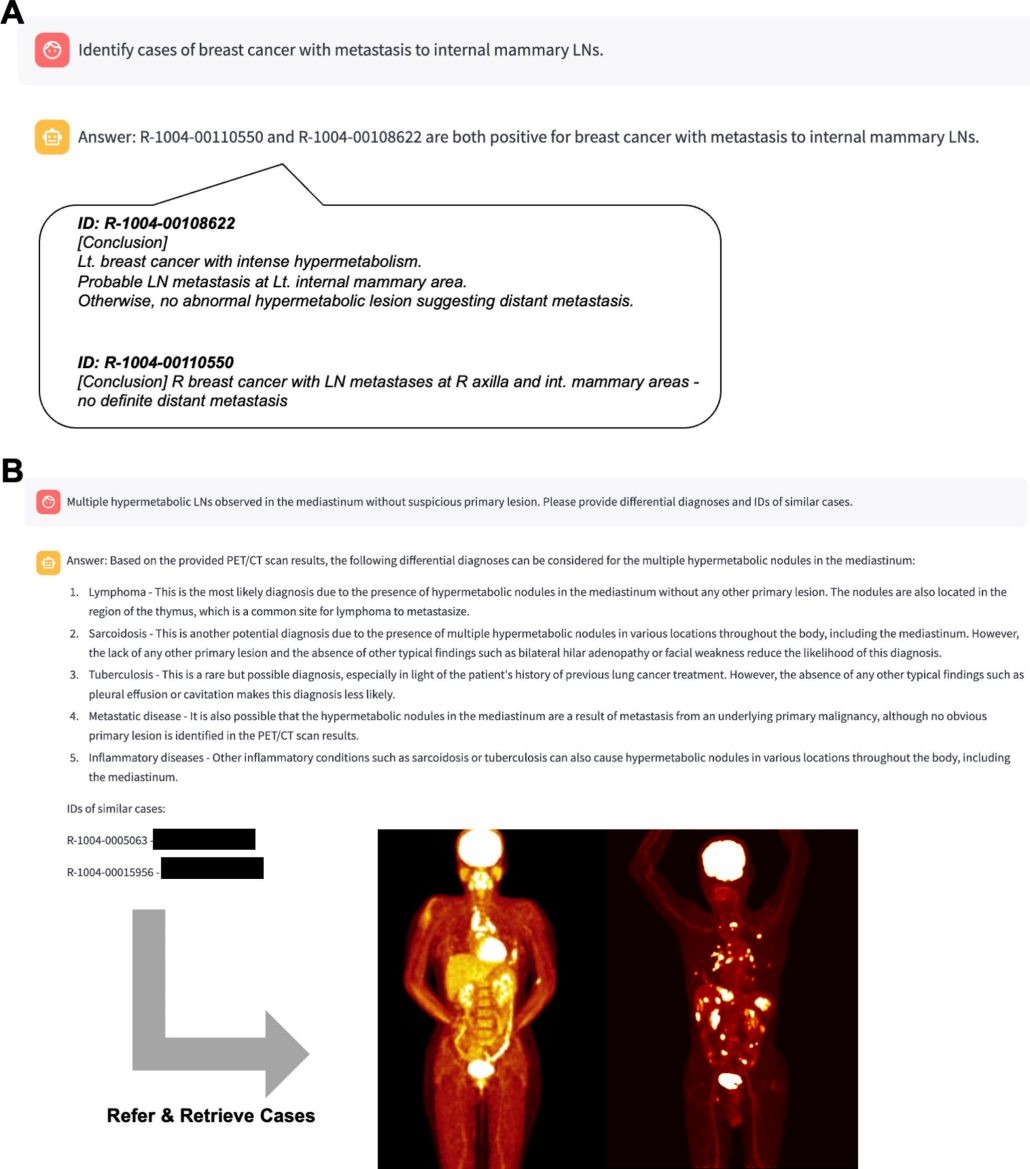
Examples of Chatbot Responses to Queries. (**A**) An example case displays an instance of the chatbot’s capability to accurately identify and present relevant cases in response to a user query about breast cancer with metastasis to internal mammary lymph nodes. It highlights the capacity to navigate a vast database of previous reading reports to identify relevant cases. (**B**) An example of the utility of system in generating differential diagnoses is displayed. This is demonstrated through the chatbot’s response to a query, where it offers a detailed list of potential diagnoses along with reference identifiers. As an example, by employing identifiers within the PACS system (in this example, we used deidentified information), prior imaging cases could be referenced for understanding cases and supporting decision making

Using the prototype chatbot, we tested its efficacy in identifying cases pertinent to specific user queries. A notable instance involved the chatbot’s response to the query, “*Identify cases of breast cancer with metastasis to internal mammary lymph nodes*,” where it proficiently located and presented relevant cases from the database of prior reading reports (Fig. 3A) (More examples are presented in Supplementary Video 1). This example demonstrates how clinicians or trainees could rapidly find comparable cases for reference, potentially aiding diagnostic reasoning or educational purposes. The retrieved cases included key details from prior reports, allowing users to cross-reference imaging findings, disease progression, and final outcomes in patients with similar clinical scenarios. Additionally, we evaluated the chatbot’s functionality in offering differential diagnoses by leveraging its integration with LLM. This was exemplified in a scenario where the chatbot was tasked to provide differential diagnoses for the condition described as “*Multiple mediastinal lymph nodes with increased FDG uptake without an identified primary site*.” The chatbot responded with a detailed list of differential diagnoses, accompanied by reference identifiers, thus enabling medical professionals to quickly locate and compare relevant case histories, imaging findings, and clinical outcomes (Fig. 3B).

Taken together, these examples underscore the potential for integrating real-world historical data into the decision-making process. By referencing prior PET reports through the RAG framework, clinicians receive contextually enriched insights, which can be especially valuable for less common clinical presentations. This improved retrieval and diagnosis suggestion process highlights a practical way to apply generative AI tools in nuclear medicine practice, where rapid access to similar cases and differential diagnoses can benefit patient care.

### Evaluating appropriateness for case querying and diagnosis suggestion using LLM with RAG

**Fig. 4:**
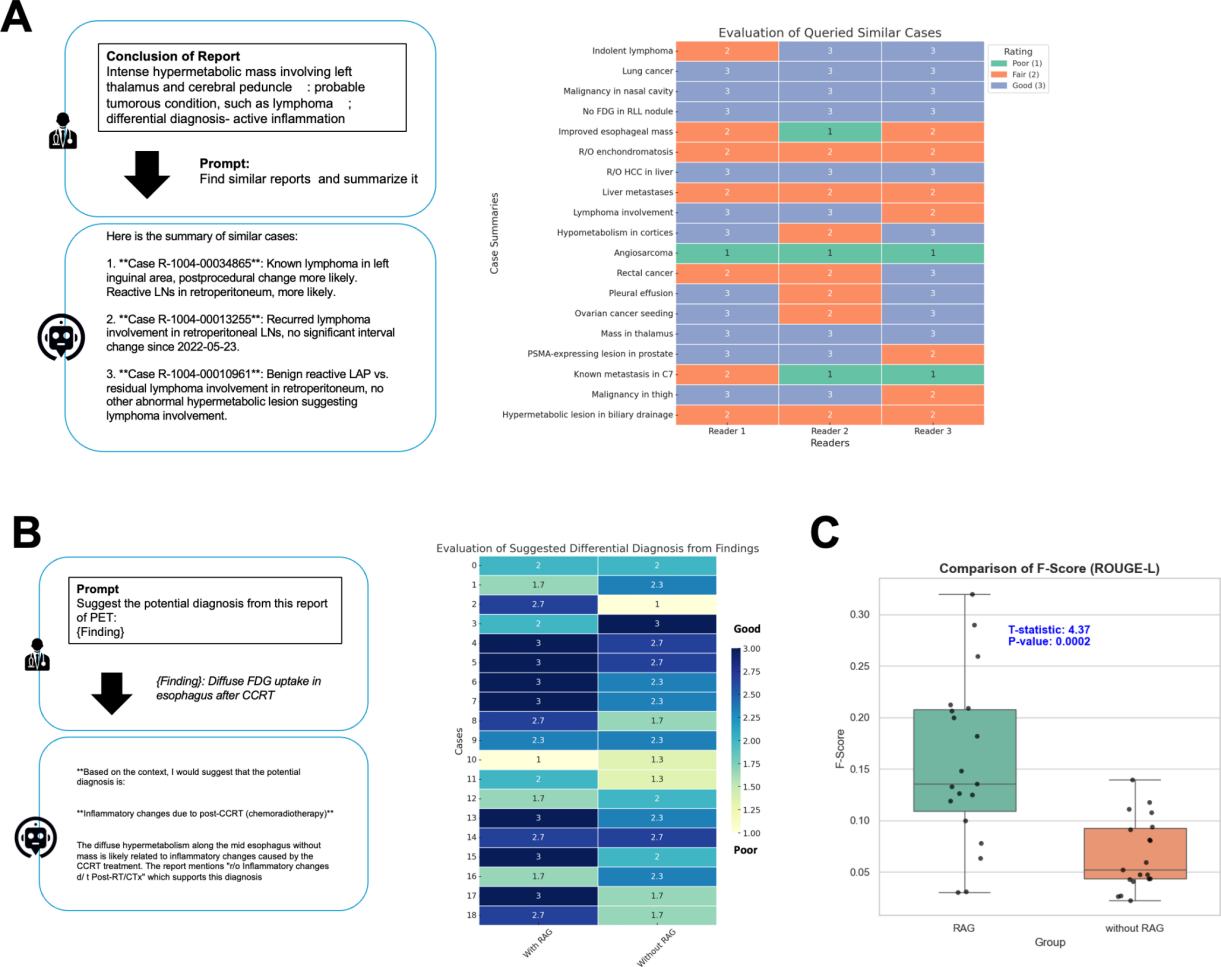
Evaluation of Appropriateness Scores by Nuclear Medicine Physicians. (**A**) The appropriateness of querying similar cases was assessed. Using a conclusion text to generate the prompt “find similar reports and summarize it,” the system retrieved results. For specific reports, 16 out of 19 (84.2%) were appropriately identified, with all three readers rating these as better than ‘Fair’ in relevance. (**B**) The appropriateness of potential diagnoses for specific findings was evaluated. Using specific finding texts to generate prompts for suggesting potential diagnoses, the responses of system were assessed. Medical relevance and appropriateness of the suggested potential diagnoses were evaluated by readers. The system without RAG was also assessed, and the performance of the LLM with and without RAG was represented as a heatmap. The results indicated that the LLM with RAG showed significantly better appropriateness scores (*p* < 0.05). (**C**) The ROUGE-L F-score was used to quantitatively evaluate the alignment between generated conclusions and reference conclusion texts from finding descriptions. The RAG framework demonstrated significantly higher scores compared to the LLM without RAG (0.16 ± 0.08 vs. 0.07 ± 0.03, *p* < 0.001)

In addition, the appropriateness scores evaluated by nuclear medicine physicians were assessed for two different simulated tasks: querying similar cases and suggesting potential diagnoses from specific findings. Firstly, for the similar cases queried by specific reports, 16 out of 19 (84.2%) were appropriately identified, with all three readers rating these as better than ‘Fair’ in relevance (Fig. 4A). Furthermore, the appropriateness of potential diagnoses for specific findings was evaluated, with 15 out of 19 (78.9%) cases receiving a better than ‘Fair (2)’ grade from all readers for the suggested potential diagnoses. To compare the performance of the LLM with and without RAG, the Wilcoxon rank sum test was conducted. The LLM with RAG showed significantly better appropriateness scores compared to the LLM without RAG (W = 226; *p* < 0.05) (Fig. 4B). In addition to the appropriateness assessed by physicians’ scores, the conclusions generated using findings with and without the RAG framework were quantitatively evaluated. The ROUGE-L F-score, which measures how well the generated conclusion from findings captures the reference conclusion text, was significantly higher for the RAG framework compared to the LLM without RAG (0.16 ± 0.08 vs 0.07 ± 0.03, *p* < 0.001; Fig. 4C).

## Discussion

In this study, we have explored the integration of LLMs into the PET imaging reporting process, presenting a novel prototype chatbot based on RAG capable of retrieving relevant cases and offering differential diagnoses based on specific user queries. This LLM with RAG represents a feasibility for medical purposes in nuclear medicine imaging field, particularly by incorporating contextual understanding from previous PET imaging reports to respond to queries from nuclear medicine physicians. This approach marks a departure from simple chatbot functionalities, introducing a system that integrates with the clinical workflow to provide contextually relevant information and insights. This proof-of-concept not only validates the utility of LLMs in enhancing the PET reporting process but also underscores the potential of AI-assisted tools to augment diagnostic accuracy and clinical decision-making in nuclear medicine.

The RAG model combines the strengths of information retrieval and generative AI to offer precise and informative answers to complex medical queries. It works by first retrieving relevant documents or data points from a vast database—in this case, a collection of PET imaging reports. Following this, the model uses the retrieved information as a context to generate responses that are not only relevant but also enriched with the specificity and detail required for decision-making. This method allows the system to provide answers that are deeply informed by historical cases and existing medical knowledge, thereby supporting physicians in diagnosing and managing patient care with a higher degree of accuracy and confidence. The introduction of RAG not only reduces the risk of hallucinations but also enhances the accuracy of responses by grounding them in specialized, domain-specific data. This is particularly important in PET reporting, where the complexity and specificity of the information require expertise-driven answers. RAG provides a viable solution for effectively applying LLMs to such specialized areas, ensuring more reliable and contextually appropriate outputs. In contrast to earlier language models that concentrated on singular tasks [13–15], models based on the RAG framework with LLMs can handle diverse queries and produce varied outputs. The RAG model, distinct from LLMs that rely solely on their pre-trained datasets, actively incorporates pertinent historical information during its response generation. Primarily, employing LLMs like ChatGPT or Gemini directly is constrained by their inability to access individual center databases, which restricts their reference to prior cases and clinical outcomes. In this regard, a previous study demonstrated that RAG applications can enhance domain-specific decision-making when using LLMs in medical fields, whereas querying and retrieving specific cases to reference previous outcomes in nuclear medicine imaging are specialized tasks addressed by our work [16]. Moreover, due to stringent regulations concerning clinical data and privacy, the transfer of clinical records to external AI servers is considered highly sensitive and is inherently prohibited in numerous healthcare institutions [17, 18]. In this context, implementing a LLM with RAG framework that utilizes PET reading reports could address these challenges by facilitating the application of real-world data in each hospital, while also avoiding the various data-related regulatory constraints. Although tested in a single-center study, this approach is tailored to individual institutions rather than serving as a universal model for all hospitals. In other words, implementing LLM with RAG frameworks to retrieve data specific to each hospital could improve responses to questions directly related to that data. In addition, this feature is especially beneficial in specialty fields like nuclear medicine, where insights drawn from previous cases are helpful for informed decision-making in current clinical scenarios.

In this study, we evaluated the performance of LLM-based answers for two tasks: querying similar cases and suggesting potential diagnoses based on previous reports. The similar case retrieval demonstrated good performance, correctly identifying similar cases in nearly 90% of instances. The use of the sentence transformer in our retrieval method of RAG provides an advantage in handling PET reports as unstructured text data, which is common in large-scale hospital settings. Unlike traditional query systems that require structured tagging, our approach allows for effective data retrieval without the need for extensive pre-processing, making it more adaptable and practical for real-world clinical applications. However, the system showed limitations with rare cases; for example, it failed to appropriately retrieve a case of scalp angiosarcoma due to its rarity. In this case, we could consider incorporating a database specifically labeled with rare cases and implementing a weighting system to prioritize their retrieval during queries. Addressing the retrieval of rare case-related data is a crucial aspect of applying LLMs in medical fields. Managing a database enriched with rarity information could significantly enhance the performance of LLMs with RAG, particularly in PET reporting, by improving their ability to handle uncommon and complex cases effectively [19]. We also assessed the use of RAG for generating answers. By leveraging contexts from previous PET reports, RAG provided reliable and medically relevant responses. In particular, during the generation of potential diagnoses, RAG could reference previous cases, which helped readers perceive the answers as reliable and relevant, mitigating the hallucination effect—a common issue with LLMs in medical applications [20, 21]. Despite the positive results, the system with RAG has limitations, especially with rare cases, and the potential diagnoses could be influenced by the contexts of queried cases, reducing the number of suggested diagnoses. Nonetheless, the ability of RAG to reference relevant cases that clinicians and readers can review adds a crucial layer of validation, reducing the potential risks associated with noise and complex multi-disease scenarios. This approach distinguishes it from the direct application of LLMs for PET reading-related questions. Additional optimized methods for using RAG to identify rare cases and incorporate more context will enhance the system’s performance. In addition, while our evaluation relied on expert judgment as the gold standard, we acknowledge the inherent subjectivity in human assessments, which may impact reproducibility. To address this, we have provided the prompts used in Supplementary Table 1, allowing for testing across various LLM systems. Future studies should incorporate objective metrics and more diverse, representative datasets to further enhance the generalizability and robustness of our approach.

The application of our system extends beyond diagnostic support, serving as a valuable educational resource. By facilitating access to similar cases, it enables medical practitioners and trainees to explore diverse clinical scenarios, thereby enhancing their diagnostic skills and understanding of nuclear medicine [4, 22]. Furthermore, the ability of this system to reference previous cases when providing differential diagnoses enriches the educational content with practical, real-world examples, fostering critical thinking and decision-making skills among trainees.

One potential application of this system is its ability to correlate imaging findings with follow-up clinical results, including final diagnoses and clinical outcomes because the RAG LLM can reference previous reports. These previous references allow readers to find similar cases and trace their future clinical outcomes or final diagnoses. By integrating historical data-driven context into the imaging interpretation process, the system offers an opportunity to provide a holistic view of the clinical journey of similar cases, from imaging to final outcome [23, 24]. This comprehensive approach facilitates a more nuanced understanding of the potential implications of specific imaging findings, guiding physicians in crafting PET imaging interpretation that are informed by both the current condition and comparable past cases. The insights derived from this analysis are invaluable for informing differential diagnosis, predicting patient outcomes, and even anticipating potential complications. Such insights are crucial for bridging the gap between imaging findings and patient management strategies, ultimately contributing to improved patient care.

However, the study also acknowledges certain limitations, including the inherent risk of generating inaccurate information (hallucinations) and the current model’s reliance on textual data [20, 21]. Additionally, due to limitations in retrieval performance, the system showed poor appropriateness score in retrieving rare cases and their related potential diagnosis. This affects the overall performance and quality, as experienced physicians would find the system most useful for rare or atypical cases. To address this, using better LLM models that allow for a larger number of tokens and can reference more previous reports simultaneously could mitigate these issues. However, this requires further study and development. Additionally, while our RAG approach avoids the pitfalls of overfitting through the use of pre-trained language models without additional training, it is important to recognize the limitations inherent in the database composition. The variability in disease prevalence across different hospitals may impact the performance of similar case retrieval, potentially limiting the generalizability of our findings. Further studies involving more diverse and representative datasets are necessary to validate and enhance the robustness of our tool. A larger, multicenter study is required to validate the approach across different clinical settings, given the variations in PET indications and disease prevalence across centers. These differences could impact the model’s performance. However, this approach leverages LLMs tailored to individual hospital settings through RAG without requiring complex LLM training, demonstrating its potential utility in this report. In addition to the challenges and future perspectives, one of the future challenges, exploring the integration of multimodal data, such as combining visual and textual analysis, are identified as essential steps forward. This future direction promises not only to mitigate the limitations but also to further enrich the system’s utility by providing a more holistic approach to medical query answering and decision support.

## Conclusion

In conclusion, our suggested AI framework affirm the transformative potential of AI-assisted tools in nuclear medicine, particularly in the context of PET imaging report analysis. The integration of an RAG LLM with a comprehensive PET imaging report database demonstrated feasibility for use in real-world clinical routines in nuclear medicine, particularly for imaging interpretation and reporting. This approach enhances the workflow of nuclear medicine physician and relevance of PET report generation, possibly supporting decision-making and providing educational benefits. It underscores the potential role of AI in improving the quality and efficacy of medical care within nuclear medicine. Furthermore, as we look to the future, the development of better LLM and multimodal models stands as a pivotal next step in overcoming current limitations and fully realizing the benefits of AI in medical imaging. This proof-of-concept study and proposed framework demonstrated the feasibility of using LLMs in the clinical routine of nuclear medicine, particularly by leveraging large report databases and showed promise for improving diagnostics, education, and patient management.

## Electronic supplementary material

Below is the link to the electronic supplementary material.

Supplementary Material 1

Supplementary Material 2

## Data Availability

Due to personal information protection policies, the complete datasets of reading reports are not available outside the hospital server. Sample data are included in the supplementary materials and their related contents can be provided by the corresponding author upon reasonable request.
